# Correlates of Physical Activity Participation among Individuals Diagnosed with Cancer: An Application of the Multi-Process Action Control Framework

**DOI:** 10.3390/ijerph20054345

**Published:** 2023-02-28

**Authors:** Allyson Tabaczynski, Kelly P. Arbour-Nicitopoulos, Ryan E. Rhodes, Catherine M. Sabiston, Linda Trinh

**Affiliations:** 1 Faculty of Kinesiology and Physical Education, University of Toronto, Toronto, ON M5S 2W6, Canada; 2 School of Exercise Science, Physical and Health Education, University of Victoria, Victoria, BC V8W 2Y2, Canada

**Keywords:** physical activity, intention formation, intention translation, action control, Multi-Process Action Control Framework, cancer, behavior change

## Abstract

Background: The purpose of this study was to test Multi-Process Action Control (M-PAC) processes as correlates of physical activity (PA) intention formation and translation (i.e., action control) in individuals diagnosed with cancer. Methods: This study was a cross-sectional survey, completed from July to November of 2020 during the COVID-19 pandemic. PA and M-PAC processes were self-reported using the Godin Leisure-Time Exercise Questionnaire and questionnaires for reflective (instrumental/affective attitudes, perceived opportunity/capability), regulatory (e.g., goal-setting, planning), and reflexive processes (habit, identity). Separate hierarchical multinomial logistic regression models determined correlates of intention formation and action control. Results: Participants (*n* = 347; M_age_= 48.2 ± 15.6) were primarily diagnosed with breast cancer (27.4%) and at a localized stage (85.0%). Most participants intended to perform PA (70.9%), yet only 50.4% met guidelines. Affective judgements (*p* < 0.001) and perceived capability (*p* < 0.01) were significantly associated with intention formation. Preliminary models indicated employment, affective judgements, perceived capability, and self-regulation to be significant (*ps* < 0.05) correlates of action control, but in the final model, only surgical treatment (*p* = 0.02) and PA identity (*p* < 0.001) were significantly associated with action control. Conclusion: Reflective processes were associated with PA intention formation, while reflexive processes were associated with PA action control. Behavior change efforts for individuals diagnosed with cancer should extend beyond social-cognitive approaches to include regulatory and reflexive processes of PA behavior (i.e., PA identity).

## 1. Introduction

Physical activity (PA) has consistently shown positive effects on the physical and mental health of individuals diagnosed with cancer, resulting in improved survival rates, physical functioning, and quality of life, and decreased fatigue [1,2,3]. These benefits have prompted the development of PA guidelines for individuals diagnosed with cancer. These guidelines suggest that individuals diagnosed with cancer should meet a minimum of 150 min/week of moderate-to-vigorous PA (MVPA) for general health benefits, or three sessions/week of 30 min of aerobic and/or resistance training for cancer-specific benefits [4,5]. However, only 8% of individuals diagnosed with cancer are meeting the moderate-to-vigorous PA (MVPA) guidelines of 150 min/week when using device-based measures [6], and only 33–44% are meeting guidelines when participation is self-reported [7,8,9,10]. During the COVID-19 pandemic, PA participation rates fell even further in this population [11]. PA participation levels are suboptimal, yet these rates are disproportionate to that of intentions to perform PA [10,12,13,14]. This is evidenced by Vallerand et al. [10,14], indicating that 71% and 58% of individuals diagnosed with hematologic cancer intended to perform aerobic PA and strength training, respectively, yet less than 51% of participants successfully met guidelines for general health benefits. Additionally, tests of correlates of PA behaviors for individuals diagnosed with cancer (e.g., kidney, breast, endometrial, colorectal) have indicated intentions to be a significant correlate of PA participation; however, minimal variance in PA behavior is explained [9,15,16,17]. This phenomenon, labelled the intention–behavior gap, has been well-documented across general and clinical adult populations [18,19], and nearly half of individuals in the general adult population who intend to participate in PA fail to do so [19]. 

The social cognitive tradition, which situates intention as the central determinant of PA behaviors, is widely and predominantly used in behavioral PA research [20], yet these approaches to understanding and intervening on behavior are limited as they fail to address this persistent intention–behavior discordance [20,21,22]. In response, several approaches (e.g., health action process approach, dual-process frameworks, I-Change) have been developed to address these shortcomings [20]. The Multi-Process Action Control (M-PAC) framework is one dual-process approach, developed specifically with PA in mind, that presents a framework for understanding both PA intention formation and the translation of intentions into behavior, coined action control [23]. The M-PAC framework suggests a progressive, layered, and dynamic framework of reflective, regulatory, and reflexive processes for understanding and predicting PA action control. The M-PAC framework hypothesizes that PA intention formation is primarily a product of reflective processes, the reasoned and deliberative thought processes influencing decision making towards PA behavior. These reflective processes derive from social cognitive approaches to understanding PA and are hypothesized to include instrumental attitudes, affective judgements, perceived capability, and opportunity [23]. Regulatory processes, in conjunction with affective judgements and perceived opportunity, are hypothesized to be determinants of initial action control (i.e., PA adoption). Regulatory processes include the self-regulatory behaviors (e.g., goal-setting, action planning) that one engages in to shield intentions from competing tendencies and support uptake of the behavior. Through continuous PA participation, reflexive processes towards PA will begin to develop; therefore, these processes are hypothesized to be predictors of PA maintenance (i.e., sustained action control). M-PAC postulates that habit and identity are important reflexive processes for PA behavior change and maintenance.

While there is research supporting the central tenets and structure of the M-PAC framework in a variety of populations [24], few studies have tested this framework in cancer populations [10,14]. A cross-sectional analysis of the M-PAC framework in individuals diagnosed with hematologic cancer has provided support for the overall structure of M-PAC in predicting action control for both aerobic and resistance training [10,14]. Further, some intervention work has also been conducted utilizing the M-PAC framework to support PA and sedentary behavior change [25,26,27]; however, these studies are either in progress or in the feasibility pilot stage of evaluation. Therefore, at present, applied tests of this framework are limited by early-stage research designs. 

While the larger structure of M-PAC has received support across various populations, there are several components of M-PAC that require further investigation, particularly among cancer populations. For example, the dichotomization of traditional self-efficacy constructs into perceived capability and perceived opportunity requires further investigation. The self-efficacy construct has historically contained both perceptions of one’s ability, as well as the availability of opportunities to perform the behavior [28]. M-PAC, however, theorizes that the distinction should be made between perceived opportunity and perceived capability as both may provide meaningful independent contributions to PA intention formation and action control [23]. Perceived opportunity is thought to be a more salient predictor of behavioral enactment, given that opportunity for PA may have greater variability while capabilities may be more stable [23,28]. This hypothesis, however, has received mixed support across populations; therefore, it is theorized that perceived capability may play a greater role in predicting behavior in clinical populations that may experience greater threats to one’s ability to perform PA successfully or safely, such as individuals diagnosed with cancer [28]. 

The perceived opportunity and capability debate among clinical populations exemplifies the need to test the M-PAC framework among cancer populations specifically, and within the context of the COVID-19 pandemic. Firstly, individuals diagnosed with cancer experience a multitude of threats to their physical and mental health following its diagnosis and treatment including fatigue, anxiety, physical limitations, and fear of recurrence, all of which can impact PA behaviors as well as its determinants [29]. As such, understanding the utility and performance of M-PAC’s processes as independent correlates is needed to maximize the framework’s predictive capacity for understanding and changing PA behavior of individuals diagnosed with cancer. Secondly, the COVID-19 pandemic has introduced numerous pervasive individual, social, and environmental barriers to PA participation that may disproportionately affect those living with and beyond cancer [30,31,32]. Other works conducted during the COVID-19 pandemic among general adult populations have indicated mixed support for M-PAC processes as predictors [33,34,35] compared to pre-pandemic research. In general adult populations, Rhodes et al. [33] reported that 17.2% of Canadian adults relapsed into physical inactivity since the start of the COVID-19 pandemic, with nearly 42% of relapsers having no intention to be physically active. As such, the substantial individual and environmental barriers presented by the COVID-19 pandemic [30,31,36] have likely altered individuals’ motivation for PA. Given similar rates of relapse in cancer populations specifically [11], M-PAC’s motivational processes and general framework should be tested to inform behavior change efforts beyond the COVID-19 pandemic. 

The primary objective of this study was to (1) determine the prevalence of intention formation (i.e., intenders vs. non-intenders) and PA action control (i.e., unsuccessful intenders vs. successful intenders) in a sample of individuals diagnosed with cancer and (2) examine M-PAC’s reflective (i.e., instrumental and affective attitudes, perceived capability and opportunity), regulatory (i.e., goal-setting, planning, self-monitoring) and reflexive processes (i.e., habit and identity) as correlates of intention formation (intenders vs. non-intenders) and action control (unsuccessful intenders vs. successful intenders) categorizations during the COVID-19 pandemic. It was hypothesized that approximately 70% of individuals diagnosed with cancer would report intentions to perform PA [10]; however, only 45% would be successful in meeting PA guidelines [8,10]. It was also hypothesized that reflective processes would be associated with PA intention formation, while only ongoing reflective processes (i.e., affective attitudes and perceived opportunity), regulatory, and reflexive processes would be associated with PA action control [10,23]. 

## 2. Materials and Methods

### 2.1. Design

This study is a secondary data analysis from a previous cross-sectional online survey conducted to examine changes in PA and its association with QoL in individuals diagnosed with cancer during the COVID-19 pandemic [11]. Data was collected from July 2020 to November 2020 using REDCap and took an estimated 45 min to complete. 

### 2.2. Participants

Eligibility criteria included: (1) ≥18 years of age, (2) diagnosed with cancer of any type, (3) able to complete the study in English. Participants were recruited through community cancer organizations within Canada and the United States, a database of individuals diagnosed with cancer consenting to being contacted for research opportunities, and through Prolific (www.prolific.co) (accessed on 22 July 2020), a survey dissemination service that recruits study participants worldwide to complete online surveys.

### 2.3. Measures

#### 2.3.1. Demographic and Medical Variables

Demographic and medical variables were collected through self-report. Demographic variables included age, sex, education, employment status, marital status, ethnicity, and country of residence. General medical and behavioral variables included number of comorbidities, body mass index (BMI), smoking status, and alcohol consumption. Cancer-specific medical variables were self-reported including cancer type, spread, current cancer status, current treatment status, treatment type, time since diagnosis, and time since last treatment. These demographic, medical, and clinical variables are commonly reported in exercise oncology research [8,37,38].

#### 2.3.2. Physical Activity

PA participation was measured using an adapted version of the Godin Leisure-Time Exercise Questionnaire [39]. Participants were asked to self-report the frequency and duration of vigorous, moderate, and light aerobic PA, and resistance training in a typical week since the start of the COVID-19 pandemic. Frequency and duration were multiplied to derive total minutes/week for each category. MVPA min/week were calculated as the sum of moderate aerobic minutes/week and two times the vigorous aerobic minutes/week. Participants who met or surpassed a total of 150 min/week of MVPA were characterized as meeting aerobic MVPA guidelines. This questionnaire has been frequently used in cancer populations (e.g., [8,38]) and has reliability coefficients of 0.83 and 0.85 [39]. 

#### 2.3.3. M-PAC Variables

Reflective, regulatory, and reflexive processes towards PA participation of M-PAC were measured via self-report. For each M-PAC measure, regular PA was defined as minimum of 150 minutes/week of at least moderate intensity. Participants were provided with examples of moderate (e.g., brisk walking, easy cycling) and vigorous (e.g., running, aerobics) PA. To encapsulate motivation towards PA of all types, participants were instructed to consider resistance training as moderate intensity PA when completing M-PAC process questionnaires. 

#### 2.3.4. Reflective Processes

Attitudes (i.e., instrumental and affective) were measured independently using separate 3-item, 7-point Likert scales [40]. Utilizing the stem “Over the next 4 weeks, engaging in PA on a regular basis would be…”, participants were presented with three different adjectives representing instrumental (i.e., wise, beneficial, useful) and affective (i.e., enjoyable, exciting, pleasant) attitudes. Participants were asked to rate each the extent to which they felt each adjective on a scale from 1 (Extremely disagree) to 7 (Extremely agree). Each scale was summed independently with higher scores representing more positive instrumental and affective attitudes. 

Participants’ perceived capability and perceived opportunity to perform regular PA was measured using separate 3-item measures [41,42]. To assess perceived capability, the following statements were made: “I possess the skills to do regular PA over the next 4 weeks if I wanted to”, “I have the physical ability to do regular PA over the next 4 weeks if I wanted to” and “I am confident that I am capable of engaging in regular PA if I had to”. Perceived opportunity was measured using the following statements: “If I really wanted to do regular PA over the next 4 weeks, I would have the chance to do so”, “I lack the opportunity to do regular PA over the next 4 weeks, even if I were really motivated to do so”, and “There are places where I can do PA at home and at work if I had to”. Participants were asked to indicate on a scale of 1 (Strongly Disagree) to 5 (Strongly Agree) the extent to which they agreed with the presented statements. Scales were summed individually with higher scores indicating greater perceived capability and opportunity. These scales have been shown to have good internal consistency with composite reliability coefficients of >0.72.

Intention to perform PA in the next 4 weeks was measured using a single-item measure [43]. Participants were asked to indicate the number of days/week they intended to perform PA over the next 4 weeks. This measure of intention is aligned with M-PAC’s conceptualization of this construct being representative of a dichotomized decision to perform a behavior rather than a post-motivational measure of the strength of one’s intention [23,44]. 

#### 2.3.5. Regulatory Processes

Regulatory processes were measured using a 6-item scale developed by Sniehotta et al. [45]. Participants were presented with six statements about performing self-regulatory behaviors (e.g., planning, self-monitoring, goal-setting) and were asked to rate the degree to which they agreed with them on a scale of 1 (Strongly Disagree) to 5 (Strongly Agree). Examples of questionnaire items include: “I kept track of my PA in a diary or log over the last month”, “I set short-term (daily or weekly) goals for leisure-time PA last month”, and “If I did not reach a PA goal last month, I analyzed what went wrong”. Scores on each item were summed with higher total scores indicating greater levels of self-regulation. This measure displayed excellent internal consistency with Cronbach alphas between 0.92 and 0.95 [45].

#### 2.3.6. Reflexive Processes

The construct of PA habit was measured using the Self-Report Behavioral Automaticity Index [46], a 4-item subscale from the validated Self-Report Habit Index [47]. This measure has been validated with an alpha of between 0.70 and 0.97 and has a strong correlation to the Self-Report Habit scale. The stem “PA is something…” was used followed by four statements to assess the automaticity of PA behaviors (i.e., “I do automatically”, “I do without having to consciously remember”, “I do without thinking”, “I start doing it before I realize I am doing it”). Participants indicated the degree to which they agreed with each statement on a 1 (Strongly Disagree) to 5 (Strongly Agree) scale. Higher scores indicated more automatized habits.

PA identity was measured using the Exercise Identity Scale [48,49]. This measure consists of four statements regarding PA identity and participants were asked to rate the extent to which they agreed or disagreed with the statement on a 1 (Strongly Disagree) to 5 (Strongly Agree) point scale. Statements of PA identity included: “I consider myself someone who does regular PA”, “When I describe myself to others, I usually include my involvement in PA”, “Others see me as someone who does PA regularly”, and “Regular PA fits the way I want to live”. Higher scores indicated greater levels of PA identity. This measure resulted in Cronbach alphas ranging from 0.82 to 0.95, suggesting good internal consistency [49].

### 2.4. Statistical Analysis

All statistical analyses were conducted in R Environment for Statistical Computing (version 4.1.0). All survey responses were analyzed for careless responding using the Longstring, Mahalanobis distance, and psychometric synonyms indices [11,50,51]. Survey responses with a Longstring index ≥13 or failed reverse scoring, a negative psychometric synonym index, or a Mahalanobis distance z-score of >3 on measures of M-PAC processes were flagged as high risk of careless responding and were excluded from this analysis. To determine intention formation categorizations, participants were classified as intenders (i.e., intend to perform PA at least 3 days/week) and non-intenders (i.e., intend to perform less than 3 days/week of PA). Of the participants classified as intenders, those successfully meeting PA guidelines of ≥150 min/week of MVPA were categorized as successful intenders while those performing <150 min/week were unsuccessful intenders, to create action control groupings. Descriptive statistics were used to characterize the sample’s demographic and medical characteristics, and action control categorizations. 

All demographic, medical, and clinical variables were dichotomized. Univariate analyses included chi-square analyses and ANOVAs to determine demographic, medical, clinical, and M-PAC variables as independent correlates of intention formation and action control categorizations independently. Variables meeting a significance level of *p* < 0.10 were included in the multivariate models. Separate hierarchical logistic regression analyses were used to determine the multivariate correlates of PA intention formation and action control. A threshold of *p* < 0.10 was used as removal criteria for demographic and medical variables within the multivariate models. The intention formation model (non-intenders vs. intenders) included three blocks: Block 1 included demographic variables, Block 2 included demographic, medical, and clinical variables, and Block 3 included demographic, medical, and clinical variables and reflective M-PAC processes. Regulatory and reflexive processes were not included in this model as they are theorized to be post-intentional determinants of PA behavior [14,23]. The action control model (unsuccessful vs. successful intenders) included five blocks: Block 1 included demographic variables, Block 2 included demographic, medical, and clinical variables, Block 3 included reflective M-PAC processes, Block 4 included regulatory M-PAC processes, and Block 5 included reflexive processes. Model fit was assessed for each block using Naglekirke’s R^2^, and deviance chi-square statistics. Statistical significance was set at *p* < 0.05.

Sensitivity analyses were conducted to ensure that main analysis results were indicative of true intention–behavior discordance, rather than a minimal lapse in meeting PA guidelines [52]. In this sensitivity analysis, action control categorizations were recoded using a PA threshold of 130 min/week to represent high (i.e., ≥130 min/week) and low (i.e., <130 min/week) PA participation. Logistic regression analyses were repeated using the recoded categorizations. 

## 3. Results

**Figure 1:**
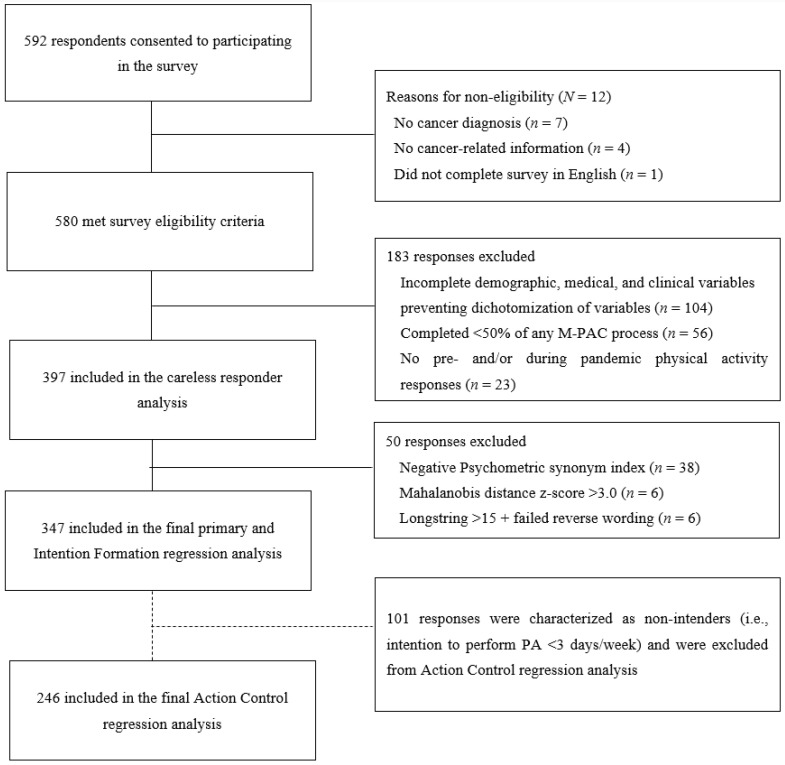
Participant flow through the study.

Survey completion and excluded responses have been previously reported [11]. Participant flow through the study is presented in Figure 1. In brief, of the 580 responses meeting inclusion criteria, 397 provided complete PA, M-PAC, and demographic and medical information. After removing responses at high risk of careless responding, the final sample in the primary analysis included 347 responses.

Demographic and medical characteristics are presented in Table 1. Participants were a mean of 48.2 ± 15.6 years of age and were primarily White (90.2%) females (69.5%) with a mean BMI of 26.7 ± 5.7 kg/m^2^. The majority of participants were diagnosed with breast (27.4%), hematologic (11.0%), or gynecologic (10.7%) cancer at a localized stage (85.0%). Participants were 90.3 ± 81.0 months from their diagnosis date at the time of completing the survey and had primarily completed primary treatments (80.4%). Participants primarily resided in the United Kingdom (37.8%), United States (26.5%), or Canada (17.9%).

### 3.1. Prevalence of Physical Activity Action Control

Participants reported performing 165.9 ± 214.6 minweek of MVPA. The majority of study participants (70.9%) intended to engage in PA ≥ three times/week. Of this, only 50.4% of participants intending to be physically active were successful in meeting MVPA guidelines (i.e., successful intenders).

### 3.2. Correlates of Intention Formation

Univariate analyses indicated cancer type (*p* < 0.05), and all M-PAC reflective processes (*p* < 0.001) were significant correlates of intention formation, while number of comorbidities and receipt of hormonal therapy met the threshold for entry (*p* < 0.10), and were therefore included in the multivariate models. No demographic variables were significant across intention formation groups. 

The results of multivariate Models 1–3 of the logistic regression examining correlates of intention formation are presented in Table 2. All medical variables in Model 2 were above the *p* > 0.10 removal threshold and therefore were removed from the model. In the final model (R^2^ = 0.33), including only reflective processes of PA, intenders were likely to have more favorable affective judgements (OR = 1.15; 95% CI = 1.08 to 1.22; *p* < 0.001) and greater perceived capability (OR = 1.24; 95% CI = 1.09 to 1.43; *p* < 0.01). 

### 3.3. Correlates of Action Control

Univariate analyses indicated months since diagnosis and months since treatment (*ps* < 0.05), and all M-PAC processes (*ps* < 0.001) were significant correlates of action control, while only employment and receipt of surgical treatment met the threshold for entry (*p* < 0.10) and were therefore included in the multivariate models. 

The results of Models 1–5 of the logistic regression examining correlates of PA action control are presented in Table 2. Model 1 included demographic variables only (*R*^2^ = 0.02). Participants who were employed were more likely to be successful intenders (OR = 0.87; 95% CI = 0.77 to 0.99; *p* = 0.04). In Model 2 (*R*^2^ = 0.04), including demographic and medical variables, successful intenders were more likely to be employed (OR = 0.58; 95% CI = 0.35 to 0.98; *p* = 0.04) and to not receive surgical treatment (OR = 0.53; 95% CI = 0.27 to 0.99; *p* = 0.05). Model 3 included demographic, medical, and reflective processes (*R*^2^ = 0.24). In this model, successful intenders were more likely to be employed (OR = 0.50; 95% CI = 0.28 to 0.88; *p* = 0.02), to have not received surgical treatment (OR = 0.46; 95% CI = 0.22 to 0.92; *p* = 0.03), and have more favorable affective judgements (OR = 1.12; 95% CI = 1.04 to 1.22; *p* < 0.01). Model 4 introduced regulatory processes into the model. In this model (*R*^2^ = 0.29), successful intenders were more likely to be employed (OR = 0.56; 95% CI = 0.30 to 1.00; *p* = 0.05), to have not received surgical treatment (OR = 0.47; 95% CI = 0.22 to 0.94; *p* = 0.04), have more favorable perceived capability (OR = 1.23; 95% CI = 1.02 to 1.50; *p* = 0.03), and have better self-regulatory behaviors (OR = 1.10; 95% CI = 1.04 to 1.16; *p* < 0.001). With the addition of reflexive processes into Model 5 (*R*^2^ = 0.35), successful intenders have not received surgical treatment (OR = 0.42; 95% CI = 0.19 to 0.88; *p* = 0.02), and have a greater identity towards PA (OR = 1.20; 95% CI = 1.08 to 1.33; *p* < 0.001).

### 3.4. Sensitivity Analysis

When recoded using a 130 min/week threshold, five additional participants (2.0% change) were categorized as successful intenders (*n* = 129). In Model 5, including demographic, medical, reflective, regulatory, and reflexive processes, only PA identity (OR = 1.04; 95% CI = 1.02 to 1.06; *p* < 0.001) was associated with PA action control using a 130 min/week threshold. In the sensitivity analysis, surgical treatment was no longer a significant correlate (*p* = 0.06). 

## 4. Discussion

This study examined the prevalence of the PA intention–behavior gap among individuals diagnosed with cancer of mixed malignancies, as well as its correlates. Aligned with our hypothesis, the majority of individuals diagnosed with cancer indicated an intention to perform PA on at least 3 days/week; however, despite these intentions, only half were meeting PA guidelines of 150 min/week of MVPA. This substantial discrepancy between PA intention and behavior adds to the growing evidence of the intention–behavior gap in this population [10,14,18,19]. Work focusing solely on intention as the proximal determinant of PA may prove inadequate in understanding and changing PA behavior for individuals diagnosed with cancer. Yet, despite this incongruence, the importance of intention formation should not be negated as behavior rarely exists in its absence [41]. As such, salient correlates of intention formation and translation must be identified to address this gap. In support of this notion, in the current study, M-PAC’s reflective processes were significantly associated with PA intention formation, but their value in predicting PA action control was limited. Reflective processes alone contributed to 33% of the variance explained in intention formation and only 18% of the variance in PA action control. The addition of regulatory and reflexive processes to predict action control contributed an additional 11% of variance explained in this model, indicating the correlates of intention formation and translation to be distinct and also supporting the overall structure of the M-PAC framework [23]. The persistent incongruence between intentions and behavior emphasizes the limited scope of social-cognitive approaches in understanding PA behavior, as well as the critical need to identify and address post-intentional determinants of PA for individuals diagnosed with cancer.

Regarding the correlates of PA intention formation, it was hypothesized according to the M-PAC framework that reflective processes (i.e., instrumental attitudes, affective judgements, perceived capability, and perceived opportunity) would be significantly associated with PA intention formation among individuals diagnosed with cancer. Our results highlight mixed support as only affective judgements and perceived capability were significantly associated with intention formation categorizations. In contrast, instrumental attitudes have been more consistently associated with PA intentions than affective judgements in previous work in this population [8,9,15,53]. The COVID-19 pandemic had a large impact on PA participation [11,30,31], and may have also altered its determinants. Many individuals diagnosed with cancer are immunocompromised, leaving them at increased risk for complications from COVID-19 [32]. This increased vulnerability may have changed their perceptions of the benefits of PA during this time. Individuals diagnosed with cancer have reported concern about contracting COVID-19 and severe complications from the infection [54]. In a mixed methods survey examining participation and perceptions of PA during the COVID-19 pandemic, nearly half of individuals diagnosed with cancer reported that PA was not a priority during the pandemic, and 61% were concerned about a return to in-person PA programming [36]. It is possible that these conflicting attitudes towards the benefits and safety of PA during the pandemic may have diminished the impact on instrumental attitudes on PA intention formation.

Furthermore, given the dramatic shift in the PA environment during the COVID-19 pandemic [30,31,36], it is interesting that perceived opportunity was not significantly associated with PA intentions. However, similar results were seen in general Canadian adult population during the COVID-19 pandemic [33]. It is possible that variance in access to PA may have been minimal given the widespread shift to primarily home-based PA settings; however, the perceptions of one’s ability to perform PA in these settings may be of greater importance for PA intention formation among individuals diagnosed with cancer. This may be especially true for individuals diagnosed with cancer who have previously attended facility-based, supervised programming. Research around the impact of perceived capability on PA behavior is mixed, and it may carry little weight in predicting PA behaviors for general adult populations; however, this construct may hold greater importance among clinical populations who may experience impaired ability to perform PA [28]. Our results highlight the importance of perceived capability on intention formation among cancer populations and supports the M-PAC’s dichotomization of the self-efficacy construct into perceived capability and perceived opportunity [23]. Future work and refinement of both dimensions of self-efficacy (i.e., perceived capability and opportunity) within the M-PAC framework is needed to obtain greater predictive capacity and specificity in intervention targets, particularly among clinical populations. Despite the current work, perceptions of available resources, time, and locations, as well as instrumental attitudes have a well-established relationship to PA intention formation and behavior in cancer populations [53,55]. As such, these constructs should continue to be targeted in understanding PA intention of individuals diagnosed with cancer, yet post-intentional factors such as regulatory and reflexive processes are necessary for intention translation.

In predicting PA action control, it was hypothesized that ongoing reflective processes (i.e., affective judgements and perceived capability), and regulatory and reflexive processes would be significantly associated with successfully translating intentions into behavior. Again, support for these hypotheses was mixed as the current study indicates the importance of affective judgments, perceived capability, regulatory behaviors, and identity as correlates for PA action control among cancer populations. While these were associated with PA action control, only identity remained as an independent correlate when all variables were included in the model. Affective judgements, perceived capability and behavioral regulation may be less predictive of behavioral maintenance than reflexive processes such as identity, aligning closely with the proposed M-PAC schematic; however, these constructs are still valuable intervention targets. 

PA identity as a strong correlate of action control is a key finding of this study. There is considerable theoretical and empirical work indicating one’s identities to be an important indicator of human behavior. Identities inform the standards of behavior with which individuals seek to act in congruence [56]. Behavior conflicting with these standards creates a negative affect which humans reflexively aim to avoid [56]. As such, an identity towards PA creates an automatic drive, or self-selection bias, towards enacting PA, as well as increases motivation to reduce discrepancies between actual behavior and one’s identity-driven standards [23,56]. Furthermore, a recent meta-analysis and narrative review conducted by Rhodes et al. [56] examining the relationship between PA identity and behavior reported a medium size effect of the association between identity and PA behavior. As such, the theoretical support for the impact of identity on behavior is further substantiated by research indicating a strong association between PA identity and participation in general adult populations, children, and adolescents [56]. 

The M-PAC framework also hypothesizes habit to be another reflexive process that is an important antecedent to PA behavior [23], yet the current work did not support this hypothesis. Support for habit as a predictor of PA is inconsistent [57], with its value being particularly diminished during the COVID-19 pandemic [33,35]. Habit is both derived and sustained from behavioral and contextual consistency, and hypothesized to trigger PA automatically when specific contextual stimuli are encountered [23,58,59]. Maintaining such automaticity during unstable and challenging conditions, such as those encountered during the COVID-19 pandemic, may be unreliable. Identity, however, is less dependent on contextual consistency as behavioral automaticity derives from the values and standards set by one’s identity that are sought to be upheld in multiple aspects of one’s life [24]. The stability of the identity construct may be indicative of its value in supporting PA action control as compared to alternative reflexive processes, such as habit, that are reliant on contextual consistency [59]. This notion has been supported by the current study, with identity as the only reflexive independent correlate of action control in the model. Therefore, the development of an identity towards PA in this population may be crucial for maintaining PA behavior among individuals diagnosed with cancer, especially when chances of behavioral lapses may be higher. 

Strategies for the development of an identity towards PA are not well known. Identity is thought to be both a product and determinant of sustained PA behavior; however, this oversimplification may prevent the advancement of intervention strategies targeting identity formation. At present, the understanding of this construct is largely confined to undergraduate and general adult populations and is limited by primarily cross-sectional designs [56], and further work is needed to identify factors that foster the development of a strong PA identity, particularly in cancer populations. However, Rhodes et al.’s [56] work also included a narrative review summarizing research on the predictors of PA identity development which may provide some insights. This review, including studies from general adult, undergraduate, and children and adolescent populations, suggested some support for reflective and regulatory processes such as commitment, perceived capability, and affective judgements/intrinsic motivation as predictors of PA identity [56]. Provided that affective judgements, perceived capability, and regulatory behaviors were correlates of PA action control and may be important factors in PA identity formation, these constructs provide valuable intervention targets for this population. 

Despite support for many of M-PAC’s constructs, the current work highlights some key deficiencies of the proposed framework in the current study. Firstly, regulatory processes are proposed to be associated with PA action control; however, in the final model, regulatory processes were not significant correlates. Similar trends have been seen in some other tests of M-PAC’s framework [60]. The M-PAC framework includes assumptions that layers of behavioral processes exert reciprocal effects on one another [23]. These bi-directional relationships between reflective, regulatory, and reflexive layers may provide a potential insight into why no reflective and regulatory processes were significantly associated with action control once reflexive processes were included in the model. The addition of PA identity may have controlled for some of the variation in lower-level processes (e.g., self-regulation). Research explicitly examining the relationship between M-PAC processes may be needed to untangle their individual effects on each other as well as behavior. Secondly, M-PAC constructs failed to account for all cancer-related outcomes in predicting PA action control, with receipt of surgical treatment indicated as an independent correlate of action control. Individuals diagnosed with cancer may experience long-term side effects, health outcomes, and barriers to PA that may not be accounted for by the M-PAC framework. Cancer-specific factors are likely to influence PA behaviors and its motivation; as such, further work investigating cancer-specific factors as moderators of M-PAC motivational processes and PA action control is warranted. 

The current study supports and expands upon the formative work testing the M-PAC framework in predicting PA intention formation and action control in hematologic cancer populations by Vallerand and colleagues [10,14]. The relatively large sample of individuals diagnosed with mixed malignancies provides further evidence in support of M-PAC’s structure in predicting and intervening on PA behaviors across various cancer types. This work is, however, limited by its cross-sectional design, self-reported PA measures, and demographically homogenous sample. Due to the recruitment approaches used that relied on self-enrollment (e.g., social media, listservs, community cancer organizations, Prolific), a response rate could not be obtained. Additionally, the homogenous sample, and the high average PA participation reported, may also contribute to the potential for a self-selection bias. Due to the PA-related content of the survey, it is possible that participants may have greater motivations towards PA compared to individuals diagnosed with cancer who did not participate in this research. Therefore, it is unclear if our findings generalize to the broader population of less motivated cancer survivors. Additionally, discrepancies between definitions in measurements of PA intentions, M-PAC processes, and meeting PA guidelines may potentially confound results. Measures of M-PAC motivational processes indicated resistance training to be considered moderate-intensity PA in the definition of regular physical activity which may influence results as determinants of aerobic PA and resistance training are distinct. Further research should consider using longitudinal designs to determine directionality of the relationships between M-PAC processes and action control. 

## 5. Conclusions

The current study strengthens the longstanding argument for theoretically driven approaches to address the intention–behavior gap in cancer populations. Current approaches to behavior change must extend beyond such deliberative thought processes within social-cognitive approaches in order to gain a better understanding of translating such intentions into prolonged behavior. The M-PAC framework represents a promising approach to addressing this divide among individuals diagnosed with cancer. Reflexive processes such as PA identity may be particularly relevant in this population and should be considered as a PA behavior change intervention target. Evaluations of the M-PAC framework in both experimental and real-world applications are needed to further refine and optimize its utility in predicting and addressing PA action control for individuals diagnosed with cancer. 

## Figures and Tables

**Table 1:** Demographic and medical characteristics of cancer survivors for intention formation (*N* = 347) and action control (*n* = 246) classifications during the COVID-19 pandemic (July–November 2020).

Variable	Intention Formation			Action Control		
	Non-Intenders (*n* = 101)	Intenders (*n* = 246)	*p*	Unsuccessful Intenders (*n* = 122)	Successful Intenders (*n* = 124)	*p*
**Demographic**						
Age						
<50 years (*n* = 182)	57 (31.3)	125 (68.7)	0.40	67 (53.6)	58 (46.4)	0.25
≥50 years (*n* = 165)	44 (26.7)	121 (73.3)		55 (45.5)	66 (54.5)	
Gender						
Male (*n* = 106)	33 (31.1)	73 (68.9)	0.67	36 (49.3)	37 (50.7)	1.00
Female (*n* = 241)	68 (28.2)	173 (71.8)		86 (49.7)	87 (50.3)	
Marital status						
Married/common law (*n* = 215)	66 (30.7)	149 (69.3)	0.48	69 (46.3)	80 (53.7)	0.25
Not married (*n* = 132)	35 (26.5)	97 (73.5)		53 (54.6)	44 (45.4)	
Education						
Some/completed high school (*n* = 65)	22 (33.8)	43 (66.2)	0.43	23 (53.5)	20 (46.5)	0.69
Some/completed higher education (*n* = 282)	79 (28.0)	203 (72.0)		99 (48.8)	104 (51.2)	
Ethnicity						
White (*n* = 313)	88 (28.1)	225 (71.9)	0.30	108 (48.0)	117 (52.0)	0.16
Other ^a^ (*n* = 34)	13 (38.2)	21 (61.8)		14 (66.7)	7 (33.3)	
Employment						
Employed (*n* = 206)	59 (28.6)	147 (71.4)	0.91	65 (44.2)	82 (55.8)	0.05 *
Unemployed (*n* = 141)	42 (29.8)	99 (70.2)		57 (57.6)	42 (42.4)	
**Medical**						
Body Mass Index (BMI)						
Under/normal weight (*n* = 147)	38 (25.9)	109 (74.1)	0.31	52 (47.7)	57 (52.3)	0.69
Overweight/obese (*n* = 200)	63 (31.5)	137 (68.5)		70 (51.1)	67 (48.9)	
Smoking behavior						
Never smoked/ex-smoker (*n* = 305)	84 (27.5)	221 (72.5)	0.12	106 (48.0)	115 (52.0)	0.19
Occasional/regular smoker (*n* = 42)	17 (40.5)	25 (59.5)		16 (64.0)	9 (36.0)	
Alcohol behavior						
Never drink (*n* = 70)	25 (35.7)	45 (64.3)	0.22	25 (55.6)	20 (44.4)	0.47
Social/regular drinker (*n* = 277)	76 (27.4)	201 (72.6)		97 (48.3)	104 (51.7)	
Number of comorbidities						
<2 (*n* = 214)	55 (25.7)	159 (74.3)	0.098 *	73 (45.9)	86 (54.1)	0.15
≥2 (*n* = 133)	46 (34.6)	87 (65.4)		49 (56.3)	38 (43.7)	
**Clinical**						
Cancer type						
Breast (*n* = 95)	19 (20.0)	76 (80.0)	0.03 *	41 (53.9)	35 (46.1)	0.44
Other ^b^ (*n* = 252)	82 (32.5)	170 (67.5)		81 (47.6)	89 (52.4)	
Months since diagnosis						
<60 months (*n* = 159)	40 (25.2)	119 (74.8)	0.17	69 (58.0)	50 (42.0)	0.02 *
≥60 months (*n* = 188)	61 (32.4)	127 (67.6)		53 (41.7)	74 (58.3)	
Current cancer status						
Cancer gone from body (*n* = 299)	88 (29.4)	211 (70.6)	0.87	102 (48.3)	109 (51.7)	0.43
Cancer still in body (*n* = 48)	13 (27.1)	35 (72.9)		20 (57.1)	15 (42.9)	
Disease Spread						
Localized (*n* = 295)	84 (28.5)	211 (71.5)	0.65	108 (51.2)	103 (48.8)	0.30
Metastasized (*n* = 52)	17 (32.7)	35 (67.3)		14 (40.0)	21 (60.0)	
**Treatment**						
Surgery						
Did not receive (*n* = 70)	19 (27.1)	51 (72.9)	0.80	19 (37.3)	32 (62.7)	0.07 *
Received (*n* = 277)	82 (29.6)	195 (70.4)		103 (52.8)	92 (47.2)	
Chemotherapy						
Did not receive (*n* = 191)	54 (28.3)	137 (71.7)	0.80	69 (50.4)	68 (49.6)	0.89
Received (*n* = 156)	47 (30.1)	109 (69.9)		53 (48.6)	56 (51.4)	
Radiation						
Did not receive (*n* = 222)	71 (32.0)	151 (68.0)	0.15	75 (49.7)	76 (50.3)	1.00
Received (*n* = 125)	30 (24.0)	95 (76.0)		47 (49.5)	48 (50.5)	
Immunotherapy						
Did not receive (*n* = 329)	94 (28.6)	235 (71.4)	0.50	119 (50.6)	116 (49.4)	0.23
Received (*n* = 18)	7 (38.9)	11 (61.1)		3 (27.3)	8 (72.7)	
Hormonal therapy						
Did not receive (*n* = 292)	91 (31.2)	201 (68.8)	0.07*	100 (49.8)	101 (50.2)	1.00
Received (*n* = 55)	10 (18.2)	45 (81.8)		22 (48.9)	23 (51.1)	
Targeted therapy						
Did not receive (*n* = 326)	96 (29.4)	230 (70.6)	0.76	117 (50.9)	113 (49.1)	0.21
Received (*n* = 21)	5 (23.8)	16 (76.2)		5 (31.3)	11 (68.8)	
Months since last treatment						
<60 months (*n* = 220)	63 (28.6)	157 (71.4)	0.90	86 (54.8)	71 (45.2)	0.04 *
≥60 months (*n* = 127)	38 (29.9)	89 (70.1)		36 (40.4)	53 (59.6)	
Treatment status						
Currently receiving treatment (*n* = 68)	22 (32.4)	46 (67.6)	0.61	26 (56.5)	20 (43.5)	0.38
Completed treatment (*n* = 279)	79 (28.3)	200 (71.7)		96 (48.0)	104 (52.0)	

^a^ Other ethnicities include Southeast Asian, Latin American, Black, South Asian, mixed ethnic background, West Asian, Arab, Chinese, Jewish and Indigenous; ^b^ Other common cancer types include hematologic, gynecologic, skin, multiple cancers, prostate, thyroid, testicular, colorectal, kidney, lung, head and neck, brain, bone; * significant at *p* < 0.10.

**Table 2:** Hierarchical logistic regression models predicting determinants of intention formation (*N* = 347) and action control (*n* = 246) among cancer survivors during the COVID-19 pandemic.

Variable	Model 1		Model 2		Model 3		Model 4		Model 5	
	OR (95% CI)	*p*	OR (95% CI)	*p*	OR (95% CI)	*p*	OR (95% CI)	*p*	OR (95% CI)	*p*
**Intention Formation** (non-intenders ^a^ vs. intenders)										
Demographic										
--	--	--	--	--	--	--				
Medical										
--	--	--	--	--	--	--				
Reflective Processes										
Instrumental Attitudes	--	--	--	--	1.04 (0.95–1.13)	0.36				
Affective Judgements	--	--	--	--	1.15 (1.08–1.22)	<0.001				
Perceived Capability	--	--	--	--	1.24 (1.09–1.43)	<0.01				
Perceived Opportunity	--	--	--	--	1.05 (0.92–1.20)	0.48				
*Model Fit*					*R^2^* = *0.33;* *χ^2^(4) = 91.41, p = 0.0*					
**Action Control** (unsuccessful ^a^ vs. successful intenders)										
Demographic										
Employment (employed ^a^ vs. unemployed)	0.87 (0.77–0.99)	0.04	0.58 (0.35–0.98)	0.04	0.50 (0.28–0.88)	0.02	0.56 (0.30–1.00)	0.05	0.58 (0.31–1.07)	0.08
Medical										
Received surgery (no ^a^ vs. yes)	--	--	0.53 (0.27–0.99)	0.05	0.46 (0.22–0.92)	0.03	0.47 (0.22–0.94)	0.04	0.42 (0.19–0.88)	0.02
Reflective Processes										
Instrumental Attitudes	--	--	--	--	1.08 (0.95–1.23)	0.25	1.05 (0.92–1.19)	0.49	1.06 (0.93–1.21)	0.38
Affective Judgements	--	--	--	--	1.12 (1.04–1.22)	<0.01	1.08 (0.99–1.18)	0.09	1.03 (0.94–1.13)	0.57
Perceived Capability	--	--	--	--	1.17 (0.98–1.41)	0.08	1.23 (1.02–1.50)	0.03	1.15 (0.94–1.42)	0.17
Perceived Opportunity	--	--	--	--	1.09 (0.94–1.26)	0.27	1.06 (0.91–1.24)	0.43	1.07 (0.92–1.26)	0.37
Regulatory processes										
Self-Regulation	--	--	--	--	--	--	1.10 (1.04–1.16)	<0.001	1.05 (1.00–1.12)	0.06
Reflexive processes										
Habit	--	--	--	--	--	--	--	--	1.00 (0.92–1.09)	0.99
Identity	--	--	--	--	--	--	--	--	1.20 (1.08–1.33)	<0.001
*Model Fit*	*R^2^* = *0.02;* *χ^2^(1) = 1.06, p = 0.30*		*R^2^* = *0.04;* *χ^2^(2) = 8.17, p = 0.02*		*R^2^ = 0.24;* *χ^2^(6) = 47.88, p < 0.001*		*R^2^* = *0.29;* *χ^2^(7) = 60.74, p < 0.001*		*R^2^* = *0.35;* *χ^2^(9) = 75.79, p < 0.001*	

^a^ Denotes reference category; ‘--’ indicates that there is no data associated with that variable within the model.

## Data Availability

The data that support the findings of this study are available from the corresponding author (LT) upon reasonable request.
